# Supplementary figures and images for: Dissecting conformational changes in APP’s transmembrane domain linked to ε-efficiency in familial Alzheimer’s disease
Source: PLoS One. 2018 Jul 2;13(7):e0200077. doi: 10.1371/journal.pone.0200077 (PMC6028146; doi:10.1371/journal.pone.0200077)

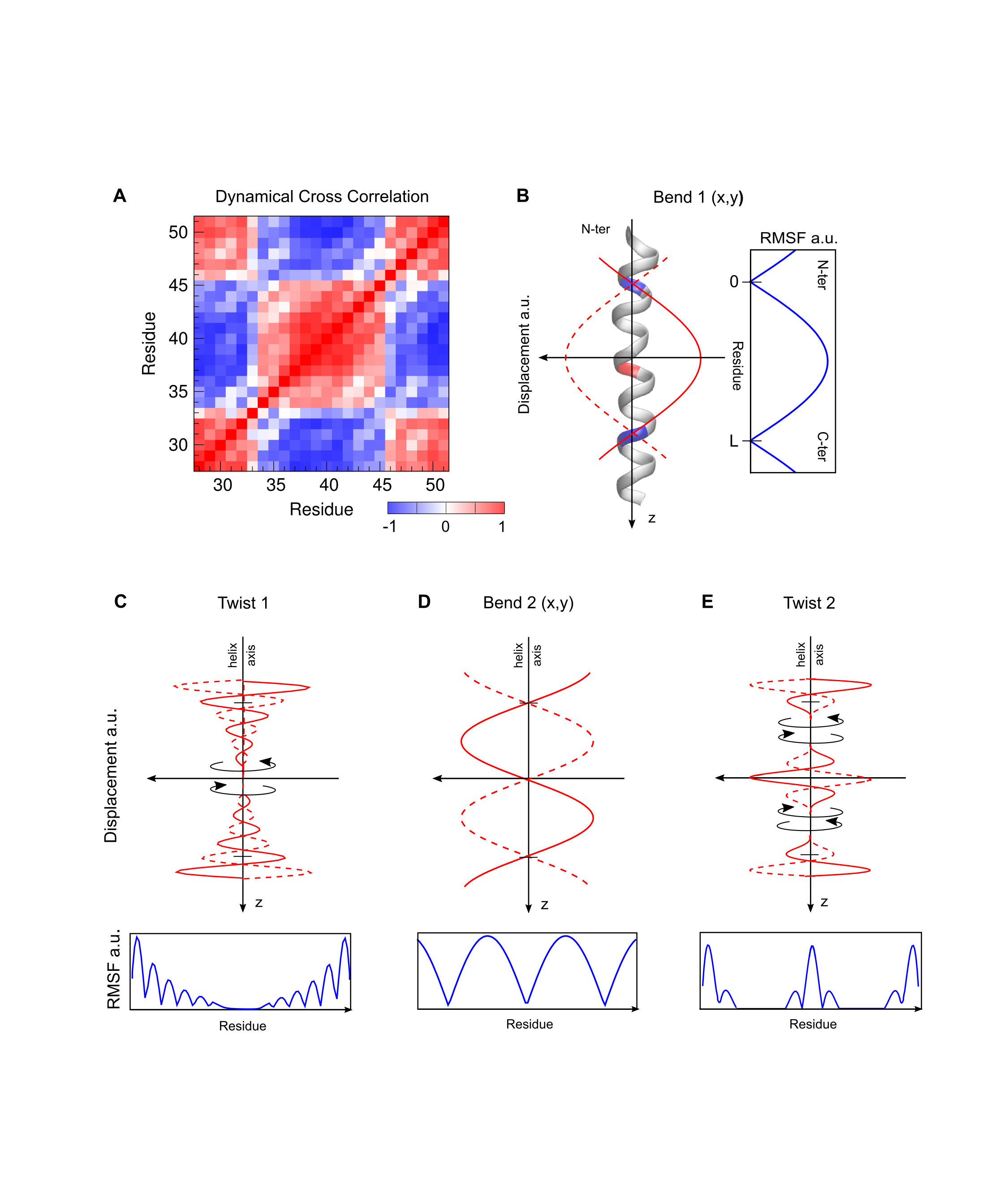

Supplement: S1 Fig — (A) Dynamic cross correlation of Cα fluctuation exemplified for the WT TMD. Deformations can be accounted for primarily by bending into the two directions (x, y) perpendicular to the helix axis (B, Bend 1 (x, y)) and twisting around the helix axis (C, Twist 1). Higher order harmonics are represented by Bend 2 (D) and Twist 2 (E). Two oscillations with a 180° phase shift are shown with full and dashed lines, respectively. The contribution of each mode to the site-specific RMSF profile is included in B-E. In each mode, flexibility and rigidity are distributed along the helix with a distinct pattern, characterized by segments with maximum (anti-nodes) and minimum (nodes) displacement. Locations of key dynamic sites are related to the node distance L. In the fundamental modes, the ideal helix bends and twists around the centre (z = L/2). The 1st harmonic provides additional centres for bending and twisting, located in TM-N (at z = L/4) and TM-C (at z = 3L/4). In this case, all residues in one segment (e.g. 0 ≤ z ≤ L/2) fluctuate anti-correlated to residues in the other segment (e.g. L/2 < z ≤ L). (TIF) [file pone.0200077.s001.tif]

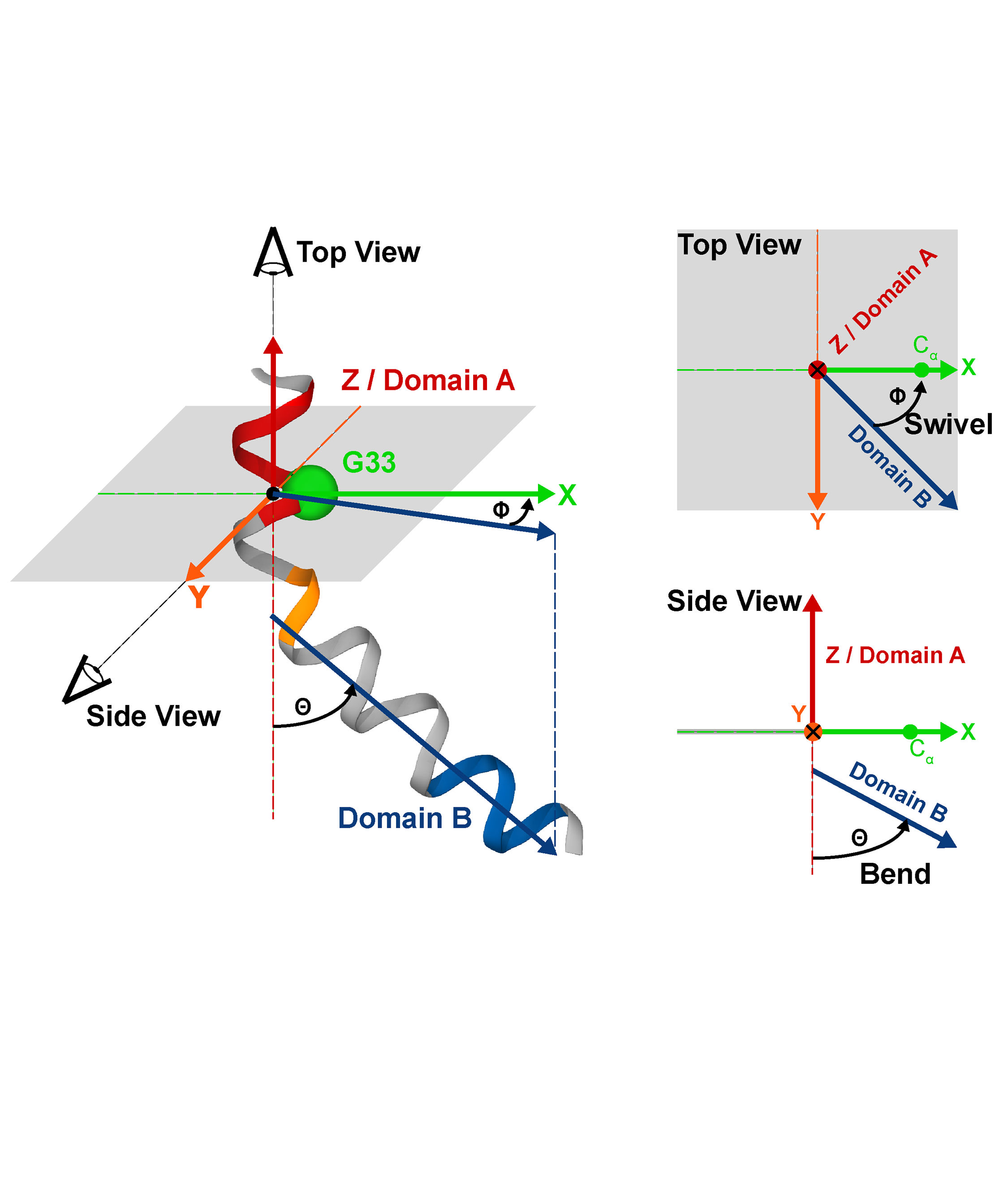

Supplement: S2 Fig — These angles describe the orientation of a helical segment in TM-C (domain B, residues I47-M51) with respect to a helical segment located in TM-N (domain A, residues A30-L34). In the first step, the helix axis of the TM-N segment is aligned with the z-axis of the coordinate system. In a second step, the helix is rotated around this axis in order to align the Cα atom of G33 along the positive x-axis. (TIF) [file pone.0200077.s002.tif]

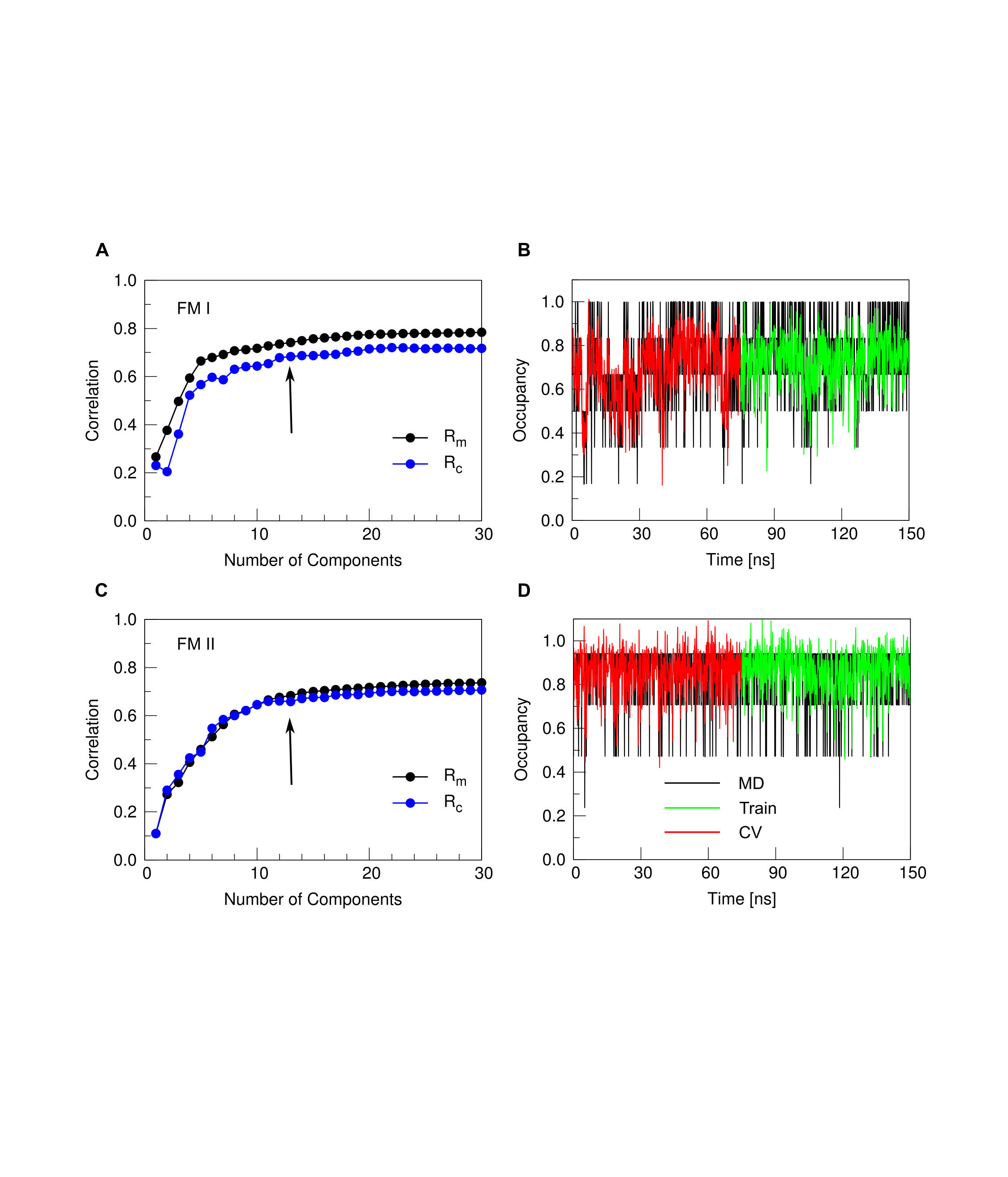

Supplement: S3 Fig — Model building and cross-validation exemplified for occupancy fluctuations in the WT TMD applied to H-bonds spanning residues G33-A42 (A, B) and residues V40-I47 (C, D), respectively. (A, C) Pearson correlation coefficients between data and model for PLS-based functional modes according to the number of PLS components calculated for the model training subset (Rm) and the cross-validation subset (Rc). (B, D) Data and model predictions for the occupancies using 13 PLS components for the MD data (black), the model training subset (red) and the cross-validation subset (green). (TIF) [file pone.0200077.s003.tif]

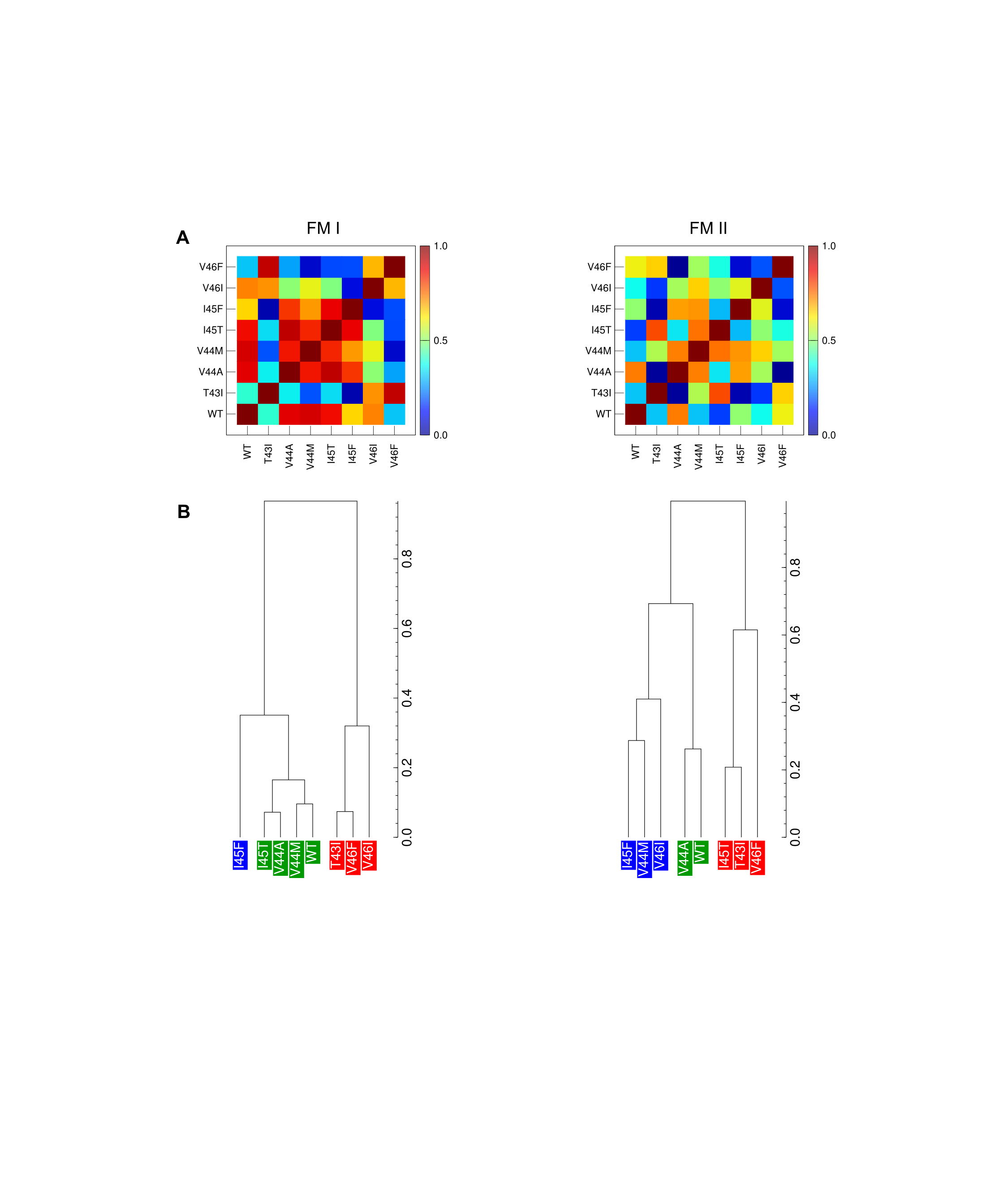

Supplement: S4 Fig — (A) Pairwise inner product for functional modes of type I (left panel) and type II (right panel), respectively. (B) Mode similarity determined from hierarchical clustering (complete linkage, zero corresponds to lowest dissimilarity). (TIF) [file pone.0200077.s004.tif]

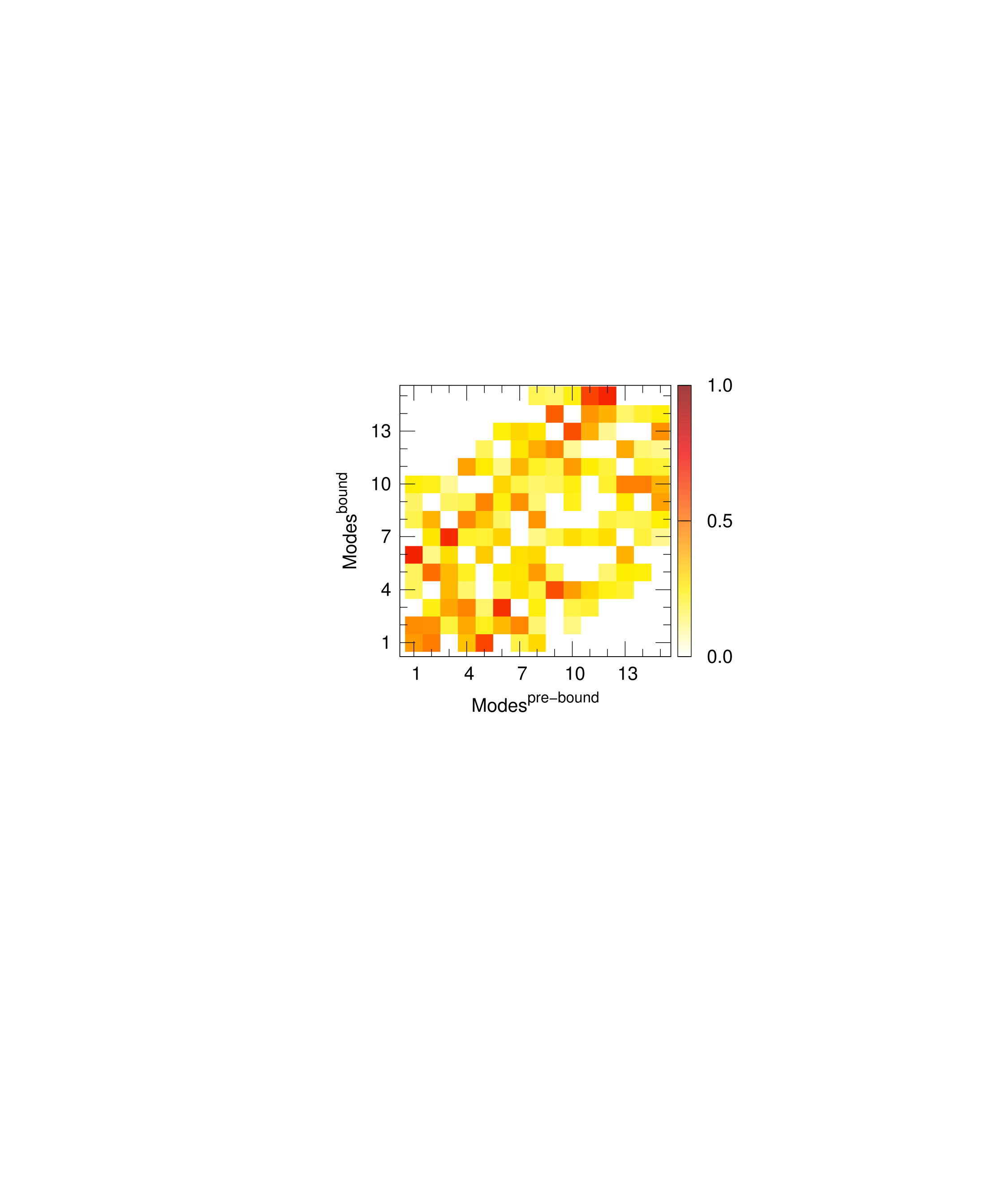

Supplement: S5 Fig — Inner product of modes of the pre-bound and bound TMD (binding model 5) exemplified for WT. Modes are numbered according to decreasing contributions to the overall MSF. Motions 1 and 2 contributing most to backbone flexibility in the pre-bound state are of less importance (rank 6 and 5) for backbone motions in the bound state. (TIF) [file pone.0200077.s005.tif]

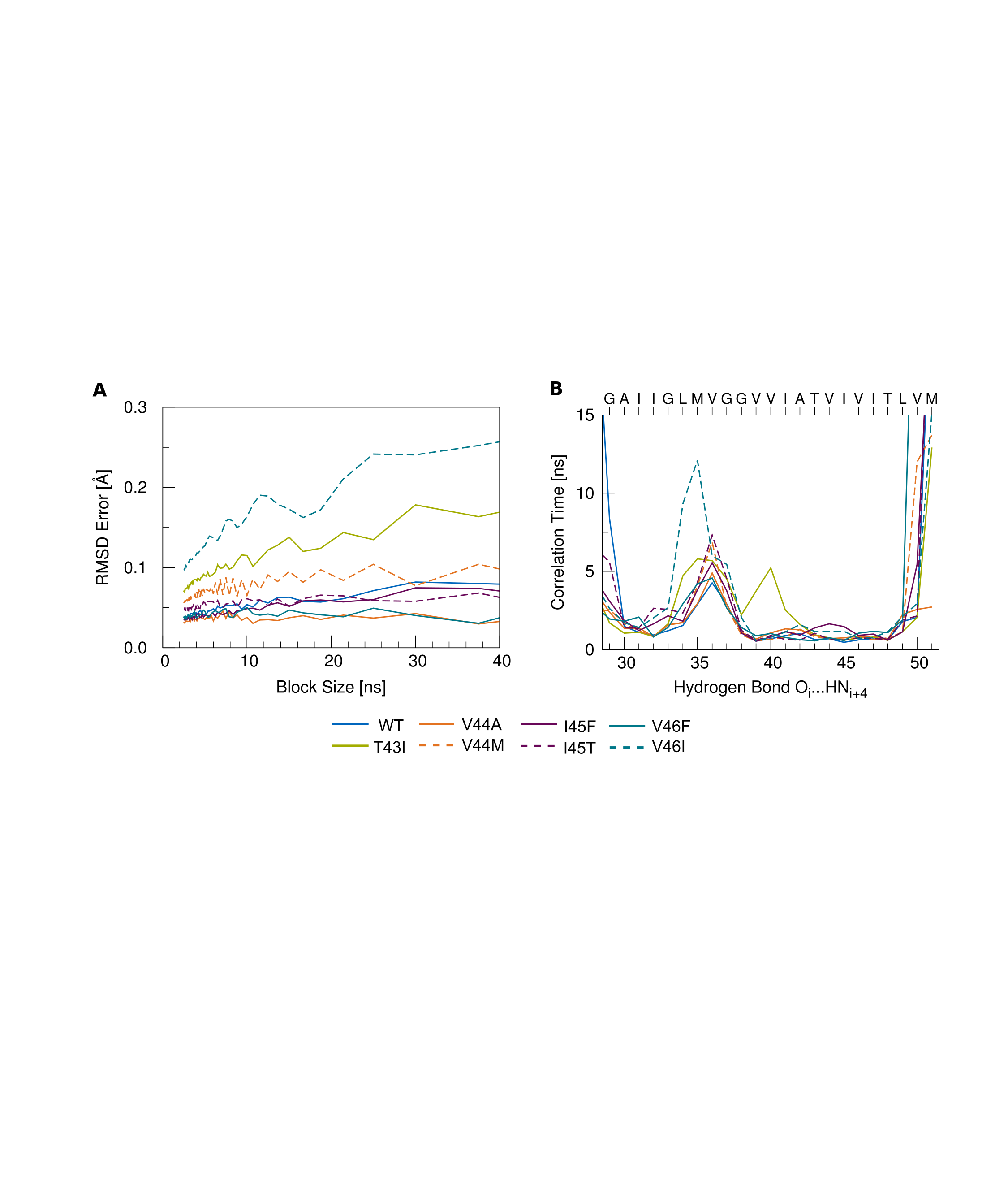

Supplement: S6 Fig — (A) Root mean-squared deviation (RMSD) of average structures with respect to the average structure (Cα atoms of residues 29–51) of the full trajectory, calculated for non-overlapping time windows of increasing length. Given is the block-averaged standard error of the mean RMSD depending on block length. The longest time to reach the plateau value is registered for the T43I mutant indicating correlated backbone fluctuation for t≤30 ns. (B) Correlation time of the length fluctuations of a-helical H-bonds calculated from the first passage of the autocorrelation through zero. The longest persistence time of H-bond fluctuations of core residues is ~12 ns. (TIF) [file pone.0200077.s006.tif]

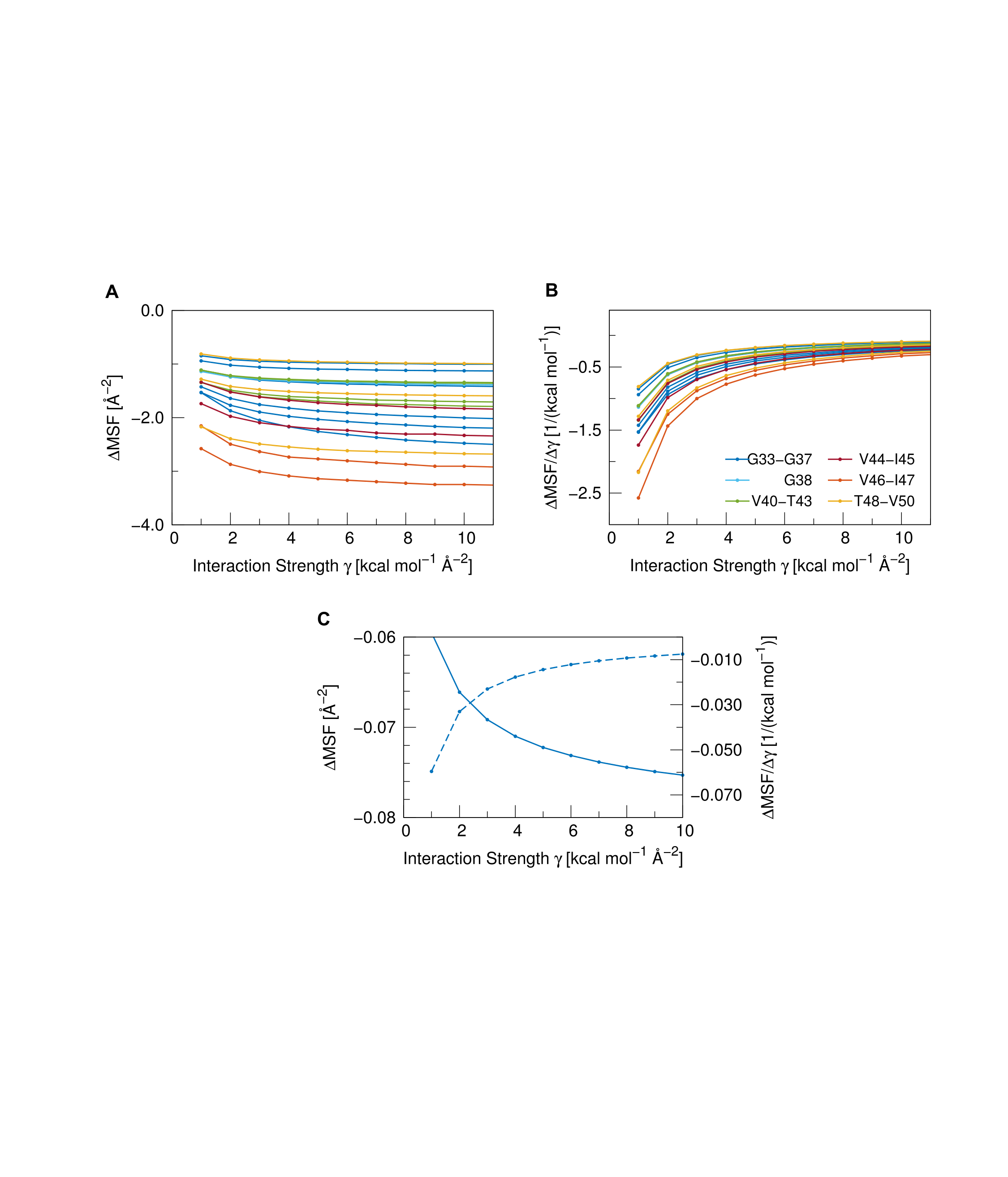

Supplement: S7 Fig — (A) Relative change of mean-squared fluctuations (MSF) with interaction strength monitored for several residues. (B) Approach to the linear regime, where the ratio ΔMSF/Δγ is expected to reach a constant value. (C) Relative change per residue (full line) and convergence (dashed line) monitored for fluctuations of backbone residues A30-V50. All calculations are based on binding model 5 (see Results). (TIF) [file pone.0200077.s007.tif]
